# Factors Associated With Resilience Among Healthcare Professionals During the COVID-19 Pandemic: A Cross-Sectional Study

**DOI:** 10.7759/cureus.25106

**Published:** 2022-05-18

**Authors:** Moroj A Aldarmasi

**Affiliations:** 1 Community Medicine, King Abdulaziz University, Jeddah, SAU

**Keywords:** mental health, healthcare workers, covid-19, stress, resilience

## Abstract

Introduction

A growing body of evidence suggests that resilience is more conducive to healthcare professionals (HCPs) adaptation and growth in the face of threats, pandemics, or other major stressful events. We aimed to measure the resilience and identify influencing factors of resilience among HCPs who have been working during the COVID-19 pandemic in Jeddah, Kingdom of Saudi Arabia.

Methodology

A cross-sectional study was performed between November 2020 and January 2021 in Jeddah. The study involved four government hospitals using an electronic self-administered questionnaire, which consisted of sociodemographic questions, the Perceived Stress Scale, and the Connor-Davidson Resilience Scale.

Results

Of the 413 participants considered in this study, only 352 were eligible. The mean resilience score of HCPs was 26±6.4. The results show significant differences across age, years of work experience, nationality, type of shift, and perceived stress score. The general linear regression model indicated that the sample population's type of shift and perceived stress score (p-value = <0.001) are statistically associated with the resilience score.

Conclusion

Attention should be paid to critical variables associated with resilience, which could help allocate scarce resources to support HCPs and retain them in the workforce.

## Introduction

There is generally a lack of preparation, coordination, and resources to deal with any pandemic, which has raised significant concerns for health and mental health [1,2]. The newest pandemic of the novel coronavirus disease (COVID-19) has gained attention worldwide [3]. The pandemic has taken over everyone's lives, and its history is constantly being rewritten [4]. Healthcare professionals (HCPs) face many stressors during their daily work, which can be aggravated even further during any pandemic [5]. Therefore, steps need to be taken to preserve the level of resilience among HCPs [5].

In psychiatry, resilience has been a concern about psychopathological processes [6]. Resilience is defined as "the ability to bounce back or recover from stress" [7] and is considered a protective factor against the development of mental disorders [8]. Resilient people can control their physical and mental health by mitigating the negative consequences of complex situations [9]. 

Research has shown that sociodemographic variables such as gender, age, and marital status affect resilience levels [10-12]. Most related studies examined relatively homogeneous populations and might not be generalizable to every society. Therefore, we conducted this study in Jeddah, Kingdom of Saudi Arabia (KSA), to assess the resilience levels, identify predictors influencing resilience, and investigate the role of perceived stress on resilience levels among HCPs in Jeddah during the COVID-19 pandemic. The results could help in planning to overcome mental health problems in future infectious disease outbreaks, help the health care facilities identify vulnerable people and proactively manage them, and to improve the resilience level of HCPs.

## Materials and methods

Study design, population, and procedure

An analytical cross-sectional study was conducted between November 2020 and January 2021 among HCPs at four Ministry of Health (MOH) Hospitals in Jeddah, KSA. The target population was HCPs working in MOH hospitals during the COVID-19 pandemic. The inclusion criterion was all HCPs, including physicians, dentists, nurses, pharmacists, radiographers, physiotherapists, dietitians, and administrators. We excluded HCPs if they had a psychiatric disease.

We selected hospitals according to their geographical distribution. At each hospital, a questionnaire was sent to all HCPs through the internal communication department. The HCPs were informed of the study's aim and objectives, and an electronic self-administered questionnaire was sent to the participant's emails through web-based software (http://www.surveymonkey.com). This software allows secure, anonymous distribution via the Internet and is password protected. A reminder was sent two weeks after the initial email. The survey structure consisted of an introduction to explain the study's purpose, a consent form, and a questionnaire.

Survey

The survey was divided into three parts. Part one covers sociodemographic characteristics, including age, gender, nationality, marital status, number of children, level of education, job position, years of working experience, monthly income, working hours per day, shift type, chronic disease, and smoking status. It also included the extent of direct interaction with COVID-19 patients and personal diagnosis of COVID-19.

Part two was the 10-item Perceived Stress Scale (PSS), a classic instrument that assesses the degree of stress experienced by participants over the past four weeks. The PSS uses a five-point Likert-scale response format (from 0 ("never") to 4 = "very often"). The score is calculated as the sum of 10 items and ranges from 0 to 40. Higher scores indicate high perceived stress [13]. The Cronbach's α of the Arabic version was 0.74 [14]. This scale is valid and has open copyright. Scores of 0-13 are considered low stress, 14-26 are considered moderate stress, and 27-40 are considered high perceived stress.

Part three was the 10-items Connor-Davidson Resilience Scale (CD-RISC), a short self-reported resilience assessment. This 10-item scale uses a 5-point response scale (0 = “not true at all,” 1 = “rarely true,” 2 = “sometimes true,” 3 = “often true,” 4 = “true nearly all the time”). The total score ranges from 0 to 40, with higher scores reflecting greater resilience [15]. Permission to use the scale was obtained. 

Data analysis

Statistical analyses were done using IBM SPSS version 23 (IBM Corp., Armonk, NY, USA). Simple descriptive statistics were used to define the characteristics of the study variables through counts and percentages for categorical variables. In contrast, continuous variables were presented by the means and standard deviations. We used an independent t-test, and one-way ANOVA with the Least Significant Difference (LSD) as a post hoc test to compare two group means and more than two groups. To correlate variables represented by means, we used Pearson's correlation coefficient. These tests were done with the assumption of a normal distribution. A general linear regression model was also used to identify significant predictors using a main-effect model. A conventional p-value <0.05 was used as a criterion to reject the null hypothesis.

## Results

A total of 413 responses were gathered, of which 25 did not finish their questionnaires, while 36 individuals were deemed to be experiencing psychiatric disorders and were therefore disqualified. The remaining 352 individuals were examined in the final analysis. The majority of the respondents were female, married, physicians, living with their families, under moderate stress, and Saudi nationals. The respondents had 36 ± 7.3 years and mean years of working experience of 11 ± 7.2. Out of all respondents, 228 individuals reported having children (64.8%). Among these, the mean number of children was 3 ± 1.3 children (N = 228; min = 1; max = 9). 

The mean resilience score was 26 ± 6.4, and the mean stress score was 19.96 ± 5.1. The majority of the respondents did not have a chronic disease (N = 283; 80.4%), but 19.6% of the respondents did (N = 69). Out of those with chronic diseases, 47.8% had hypertension (N = 33), 27.5% had asthma (N = 19), 26.1% had diabetes (N = 18), and 26.1% were obese (N = 18). Furthermore, 27.4% of all respondents had other chronic conditions, such as hypothyroidism, autoimmune diseases, cardiac disease, and osteoporosis. Other characteristics of the sample are demonstrated in Table 1.

**Table 1:** Sociodemographic Characteristics of the Study Samples

Variables	Items	Count	%
Gender	Female	222	63.1
	Male	130	36.9
Marital status	Single	91	25.9
	Married	234	66.5
	Widowed	3	0.9
	Divorced	24	6.8
Have children	Yes	228	64.8
	No	124	35.2
Living with	Family	330	93.8
	Friend	2	0.6
	Alone	20	5.7
Stress categories	Low stress	44	12.5
	Moderate stress	278	79.0
	High stress	30	8.5
Nationality	Non-Saudi	27	7.7
	Saudi	325	92.3
Highest level of education	Secondary School	4	1.1
	Diploma	69	19.6
	Bachelors	145	41.2
	Postgraduate studies	134	38.1
Job position	Physician	129	36.6
	Dentist	16	4.5
	Nurse	82	23.3
	Pharmacist	9	2.6
	Allied Health Personnel	72	20.5
	Others	44	12.5
Income per month	Less than 5000 SR	10	2.8
	From 5000 to 10000 SR	71	20.2
	11000 to 20000 SR	176	50.0
	21000 to 30000 SR	55	15.6
	More than 30000 SR	40	11.4
Smoking status	Yes	93	26.4
	No	238	67.6
	Ex-smoker	21	6.0
Deal directly with COVID-19 patients	Yes	193	54.8
	No	159	45.2
Working hours per day	less than 8 hours	14	4.0
	8 hours	215	61.1
	from 9 to 12 hours	105	29.8
	more than 12 hours	18	5.1
Usual type of shift	Morning shift	223	63.4
	Evening shift	11	3.1
	Night shift	6	1.7
	Mixed shift	112	31.8
Comorbidities (chronic diseases)	Yes	69	19.6
	No	283	80.4
Diagnosed to have COVID-19 based on lab result	Yes	54	15.3
	No	298	84.7

Table 2 shows the convergent validity between the resilience score and stress score. A moderate negative correlation was found with a correlation coefficient (r) of -0.54, which is significant at the 0.01 level. The relationship between the variables is inversely proportional. 

**Table 2:** Convergent Validity Between Resilience Score and Test Score

Correlations		Stress score
Resilience score	r	-0.549^**^
	p-value	<0.001
	N	352
**. Correlation is significant at the 0.01 level (2-tailed).		

Table 3 illustrates the correlation of independent variables (age and working experience) with the resilience score. Age had a statistically significant correlation with the resilience score (p-value = <0.001). Similarly, there was a weak positive correlation between working experience and the resilience score (p-value = 0.001).

**Table 3:** Association and Correlation of the Independent Variables with Resilience Score and Stress Score

Correlations		Resilience score
Age	r	0.207^**^
	p-value	<0.001
	N	352
Working experience in years	r	0.174^**^
	p-value	0.001
	N	352
**. Correlation is significant at the 0.01 level (2-tailed).		

Table 4 shows the association between the demographic characteristics of the respondents and the resilience score. Nationality was found to be significantly associated with the resilience score (p-value = 0.015), with non-Saudi nationals demonstrating a higher resilience score than Saudi nationals (28.85 ± 6.0 > 25.74 ± 6.4). The type of shift was also significantly associated with the resilience score (p-value = 0.014), with respondents assigned to work night shifts appearing to have the highest resilience scores (N = 6; 29.33 ± 7.3), followed by those working morning shifts (N = 223; 26.38 ± 6.6), mixed shift schedules (N = 112; 25.51 ± 6.0), and evening shifts (N = 11; 20.73 ± 3.8). 

**Table 4:** Association Between the Respondents’ Demographic Characteristics and the Dependent Variable

Variables		Total	Resilience score
Gender	Female	222	25.50 ± 6.1
	Male	130	26.78 ± 6.9
p-value			0.072
Marital status	Single	91	25.62 ± 6.5
	Married	234	25.91 ± 6.3
	Widowed/Divorced	27	27.70 ± 6.9
p-value			0.321
Have children	Yes	228	26.17 ± 6.4
	No	124	25.62 ± 6.4
p-value			0.446
Living with	Family	330	25.88 ± 6.2
	Alone	20	28.45 ± 8.2
p-value			0.185
Nationality	Non-Saudi	27	28.85 ± 6.0
	Saudi	325	25.74 ± 6.4
p-value			0.015^a^
Highest level of education	Diploma and below	73	27.16 ± 6.4
	Bachelors	145	25.27 ± 6.6
	Postgraduate studies	134	26.09 ± 6.2
p-value			0.115
Job position	Physician	129	25.59 ± 6.0
	Dentist	16	24.06 ± 6.1
	Nurse	82	25.73 ± 6.8
	Pharmacist	9	22.89 ± 6.8
	Allied Health Personnel	72	27.07 ± 6.8
	Others	44	27.09 ± 6.1
p-value			0.182
Income per month	Less than 5000	10	26.50 ± 4.5
	From 5000 to 10000	71	26.38 ± 6.2
	11000 to 20000	176	25.78 ± 6.5
	21000 to 30000	55	25.98 ± 6.8
	More than 30000	40	25.98 ± 6.2
p-value			0.972
Smoking status	Yes	93	26.46 ± 6.8
	No	238	25.73 ± 6.3
	Ex-smoker	21	26.57 ± 5.7
p-value			0.588
Deal directly with COVID-19 patients	Yes	193	25.85 ± 6.5
	No	159	26.13 ± 6.3
p-value			0.688
Working hours per day	less than 8 hours	14	26.21 ± 6.8
	8 hours	215	26.25 ± 6.4
	from 9 to 12 hours	105	25.31 ± 6.5
	more than 12 hours	18	26.39 ± 6.0
p-value			0.661
Usual type of shift	Morning shift	223	26.38 ± 6.6^A^
	Evening shift	11	20.73 ± 3.8^B^
	Night shift	6	29.33 ± 7.3^A^
	Mixed	112	25.51 ± 6.0^A^
p-value			0.014^b^
Diagnosed to have COVID-19 based on lab result	Yes	54	26.46 ± 6.7
	No	298	25.89 ± 6.3
p-value			0.543
Comorbidities (chronic diseases)	Yes	69	25.19 ± 6.5
	No	283	26.17 ± 6.4
p-value			0.256
^a^-significant using Independent t-test at <0.05 level. ^b^-significant using One-Way ANOVA Test <0.05 level. *CAPITAL letters indicate Post-Hoc multiple pairing summary indicator. Having the same letter means the same measure statistically.			

Tables 5 and 6 show the results of the one-way ANOVA with LSD post hoc test to compare the effects of nationality, type of shift, age, work experience, and stress score on the dependent variable, resilience score. The r-squared value of 0.330 (adjusted r-squared = 0.316) demonstrates a low effect size [16]. It indicates that the indicated independent variables explain 33% of the variance in the resilience score. Of these variables, the type of shift covered (F (1, 344) = [2.733], p = 0.044) and the stress score (F (1, 344 = [131.616], p = <0.001) were statistically significant. Furthermore, the general linear regression model at the <0.05 level revealed that working evening shifts (p-value = 0.008) and the mean stress scores of the sample population (p-value = <0.001) were statistically associated with the resilience score.

**Table 5:** Tests of Between-Subjects Effects

Source	Type III Sum of Squares	df	Mean Square	F	p-value
Corrected Model	4746.304^a^	7	678.043	24.210	<0.001
Intercept	4940.351	1	4940.351	176.397	<0.001
Nationality	23.254	1	23.254	0.830	0.363
Usual type of shift	229.651	3	76.550	2.733	0.044
Age	48.815	1	48.815	1.743	0.188
Working experience in years	2.893	1	2.893	0.103	0.748
Stress score	3686.167	1	3686.167	131.616	<0.001
Error	9634.429	344	28.007		
Total	251865.938	352			
Corrected Total	14380.734	351			
^a^-R Squared = 0.330 (Adjusted R Squared = 0.316)					

**Table 6:** Parameter Estimates

Parameter	B	S.E.	95% Confidence Interval		p-value
			Lower Bound	Upper Bound	
Intercept	35.848	2.506	30.919	40.778	<0.001^a^
Nationality =Non-Saudi	1.020	1.119	-1.181	3.221	0.363
Usual type of shift =Morning shift	-0.148	0.631	-1.388	1.093	0.815
Usual type of shift =Evening shift	-4.488	1.677	-7.786	-1.191	0.008^a^
Usual type of shift =Night shift	1.770	2.236	-2.627	6.168	0.429
Age	0.100	0.075	-0.049	0.248	0.188
Working experience in years	-0.024	0.074	-0.170	0.122	0.748
Stress score	-0.657	0.057	-0.769	-0.544	<0.001^a^
^a^-Significant using General Linear Regression Model (GLRM) at <0.05 level.					

## Discussion

Pandemics usually occur suddenly, and HCPs play essential roles in such situations. Psychological well-being is an essential aspect of these circumstances. We conducted this study to assess the resilience levels among HCPs and to identify influencing factors. The present findings could help policymakers streamline or calibrate standards and regulations regarding how to equip best, prepare, and support HCPs as they provide continuing healthcare services amidst a healthcare crisis like the COVID-19 pandemic.

The mean resilience score was 26, similar to that reported for Chinese HCPs [17]. Perceived stress reflects the psychological experience after self-interpretation of stressful circumstances. The present study revealed a negative correlation between stress scores and resilience scores. This finding is consistent with the results found by Karabulak and Kaya [18]. Several empirical studies also revealed the same trend of findings, where resilience was negatively correlated with poor mental health and indicators of psychiatric disorders, including depression and anxiety. Conversely, resilience was positively correlated with psychological well-being and indicators of good mental health [6,18-20]. Therefore, hospital administrators should work to relieve the stress levels of HCPs. 

We found that older age was associated with higher overall resilience, similar to a study conducted in the United States [21]. Particular attention should be paid to the mean working experience in years, and we noticed a statistically significant correlation between years of working experience with the resilience score. Another study demonstrated similar findings that resilience increased with advancing age and work experience [22]. The rationale for this has not been well studied, but there is a hypothesis that older people have been exposed to more adversity throughout their life [23]. In line with this, Minahan et al. indicated that age could be considered a protective factor among all healthcare workers amidst the pandemic as specific coping strategies would have already been in place with advancing age [24].

Another factor that significantly affected the resilience level in this study was nationality. Nationality was significantly associated with the resilience score, with non-Saudi nationals demonstrating higher resilience scores than Saudi nationals. This could be due to the use of cultural or spiritual coping and ethnic identity [25]. Other variables did not affect the resilience level in the multivariate analysis.

The present study has limitations. First, the participants in this study were limited to one city. It is recommended that future related studies be conducted in a broader setting. Cluster sampling is also recommended to guarantee that the sample is representative. Furthermore, it should be noted that reporting bias is present since a self-administered questionnaire was used to collect the data. Despite these limitations, the current findings provide vital information to address the gap in knowledge of the factors influencing the psychological effects of the pandemics and the mental health status of HCPs.

## Conclusions

Attention should be paid to factors affecting resilience levels, including age, years of working experience, nationality, type of shift, and high perceived stress. The findings of this paper could be used as a starting point for future research on the same topic. Efforts need to be initiated to strengthen resilience levels among HCPs by providing mental help and crisis management support.

## References

[1] Chevance A, Gourion D, Hoertel N (2020). Ensuring mental health care during the SARS-CoV-2 epidemic in France: a narrative review. Encephale.

[2] Aldarmasi MA, Alghamdi AH (2021). Factors influencing stress perception among healthcare workers during the coronavirus pandemic: a multi-centric cross-sectional study. IJMRHS.

[3] Xiang YT, Yang Y, Li W, Zhang L, Zhang Q, Cheung T, Ng CH (2020). Timely mental health care for the 2019 novel coronavirus outbreak is urgently needed. Lancet Psychiatry.

[4] Chaplin S (2020). COVID‐19: a brief history and treatments in development. Prescriber.

[5] Santarone K, McKenney M, Elkbuli A (2020). Preserving mental health and resilience in frontline healthcare workers during COVID-19. Am J Emerg Med.

[6] Hu T, Zhang D, Wang J (2015). A meta-analysis of the trait resilience and mental health. Pers Individ Differ.

[7] American Psychological Association (2014). The road to resilience.

[8] Shrivastava A, Desousa A (2016). Resilience: a psychobiological construct for psychiatric disorders. Indian J Psychiatry.

[9] Yang C, Xia M, Han M, Liang Y (2018). Social support and resilience as mediators between stress and life satisfaction among people with substance use disorder in China. Front Psychiatry.

[10] Purvis TE, Saylor D (2019). Burnout and resilience among neurosciences critical care unit staff. Neurocrit Care.

[11] Park JS, Lee EH, Park NR, Choi YH (2018). Mental health of nurses working at a government-designated hospital during a MERS-CoV outbreak: a cross-sectional study. Arch Psychiatr Nurs.

[12] Ang SY, Uthaman T, Ayre TC, Mordiffi SZ, Ang E, Lopez V (2018). Association between demographics and resilience - a cross-sectional study among nurses in Singapore. Int Nurs Rev.

[13] Cohen S (1988). Perceived stress in a probability sample of the United States. https://psycnet.apa.org/record/1988-98838-002.

[14] Chaaya M, Osman H, Naassan G, Mahfoud Z (2010). Validation of the Arabic version of the Cohen Perceived Stress Scale (PSS-10) among pregnant and postpartum women. BMC Psychiatry.

[15] Connor KM, Davidson JR (2003). Development of a new resilience scale: the Connor-Davidson Resilience Scale (CD-RISC). Depress Anxiety.

[16] Moore DS, Kirkland S (2007). The basic practice of statistics. https://web.ntpu.edu.tw/~hlw/Notes/bps3ed/bps_ch17.pdf.

[17] Liang Y, Wu K, Zhou Y, Huang X, Zhou Y, Liu Z (2020). Mental health in frontline medical workers during the 2019 novel coronavirus disease epidemic in China: a comparison with the general population. Int J Environ Res Public Health.

[18] Karabulak H, Kaya F (2021). The relationship between psychological resilience and stress perception in nurses in Turkey during the COVID-19 pandemic. J Nurs Res.

[19] Wu Y, Sang ZQ, Zhang XC, Margraf J (2020). The relationship between resilience and mental health in Chinese college students: a longitudinal cross-lagged analysis. Front Psychol.

[20] Klainin-Yobas P, Vongsirimas N, Ramirez DQ, Sarmiento J, Fernandez Z (2021). Evaluating the relationships among stress, resilience and psychological well-being among young adults: a structural equation modelling approach. BMC Nurs.

[21] Croghan IT, Chesak SS, Adusumalli J (2021). Stress, resilience, and coping of healthcare workers during the COVID-19 pandemic. J Prim Care Community Health.

[22] Yusefi AR, Daneshi S, Davarani ER, Nikmanesh P, Mehralian G, Bastani P (2021). Resilience level and its relationship with hypochondriasis in nurses working in COVID-19 reference hospitals. BMC Nurs.

[23] Hayman KJ, Kerse N, Consedine NS (2017). Resilience in context: the special case of advanced age. Aging Ment Health.

[24] Minahan J, Falzarano F, Yazdani N, Siedlecki KL (2021). The COVID-19 pandemic and psychosocial outcomes across age through the stress and coping framework. Gerontologist.

[25] Raghavan SS, Sandanapitchai P (2019). Cultural predictors of resilience in a multinational sample of trauma survivors. Front Psychol.

